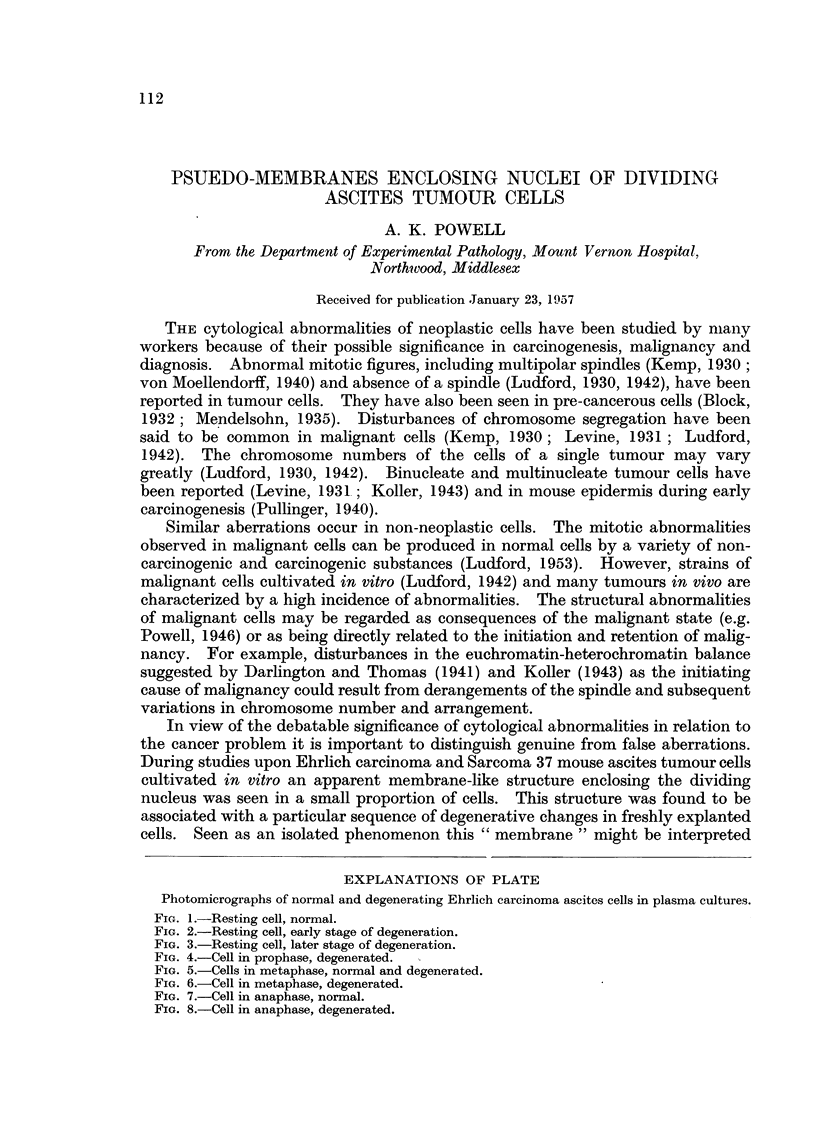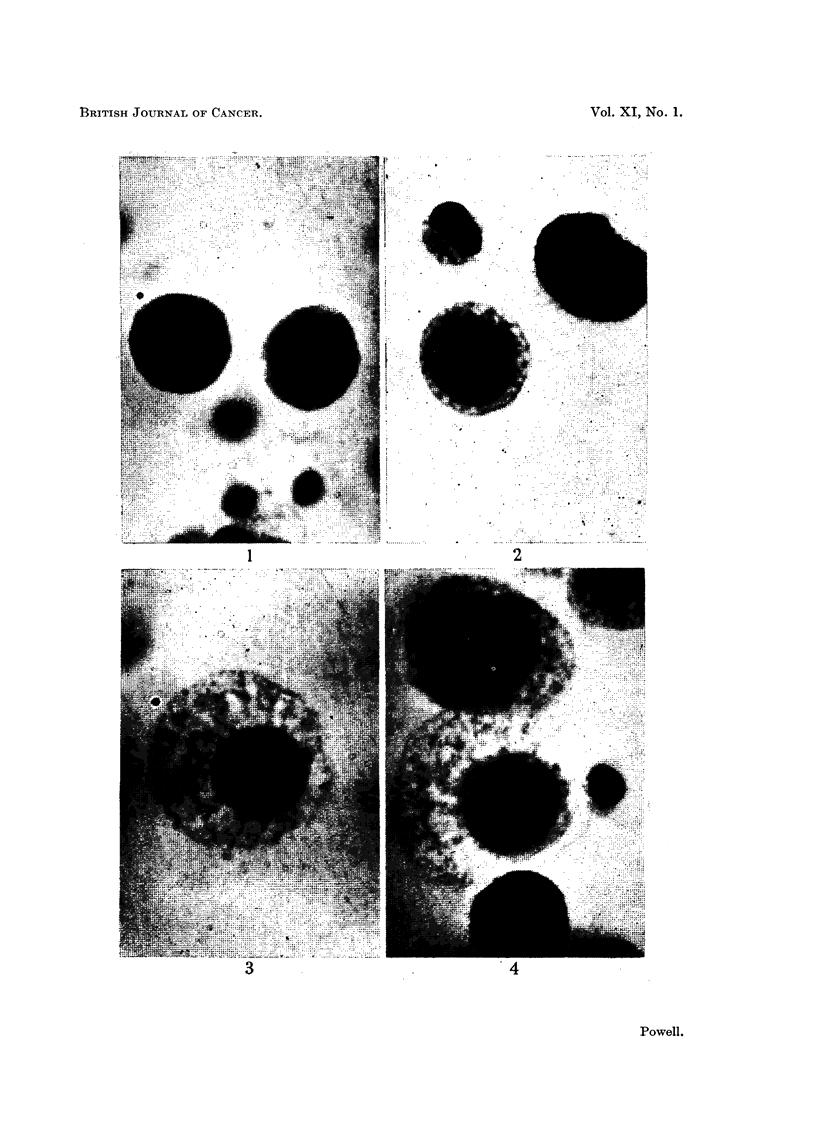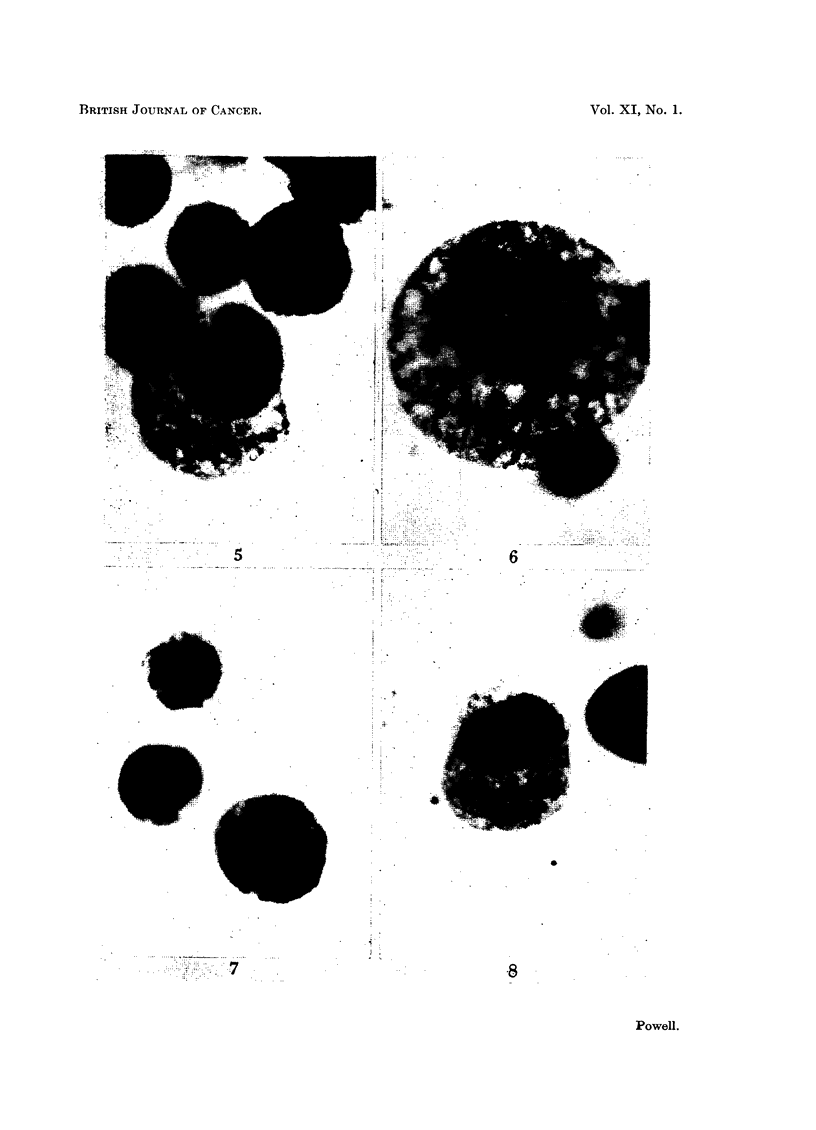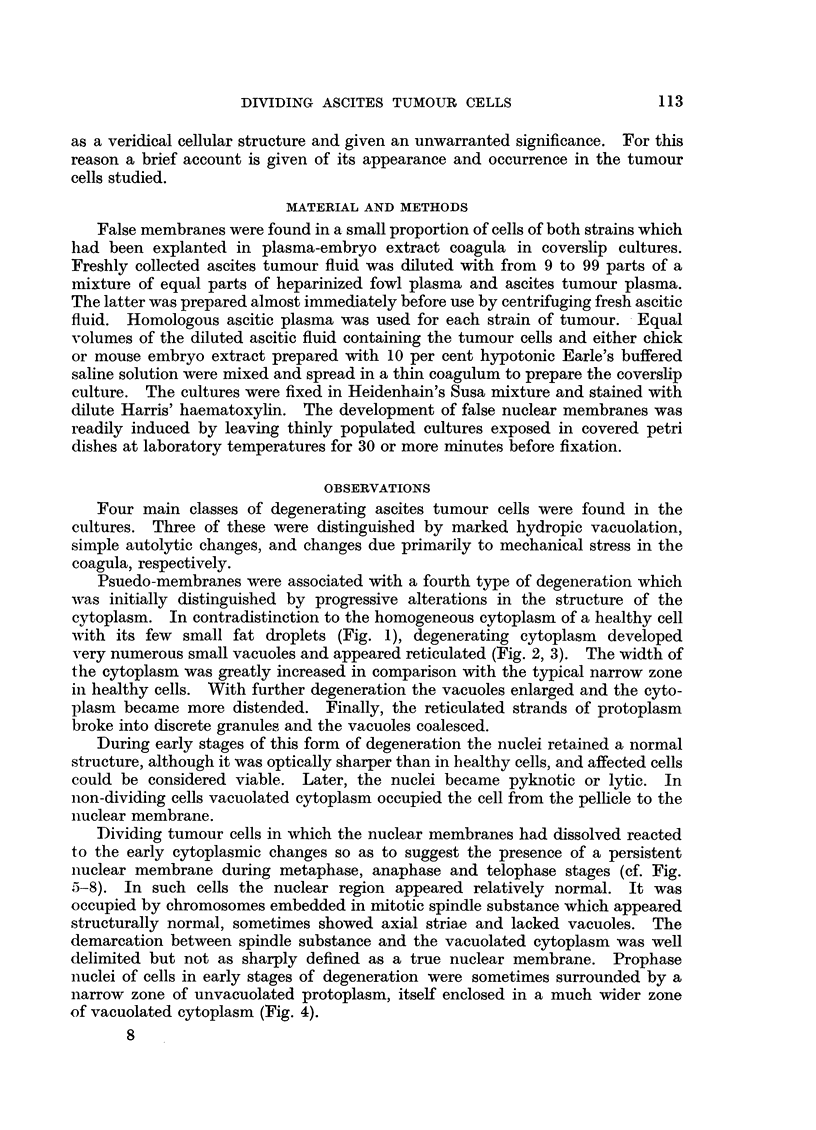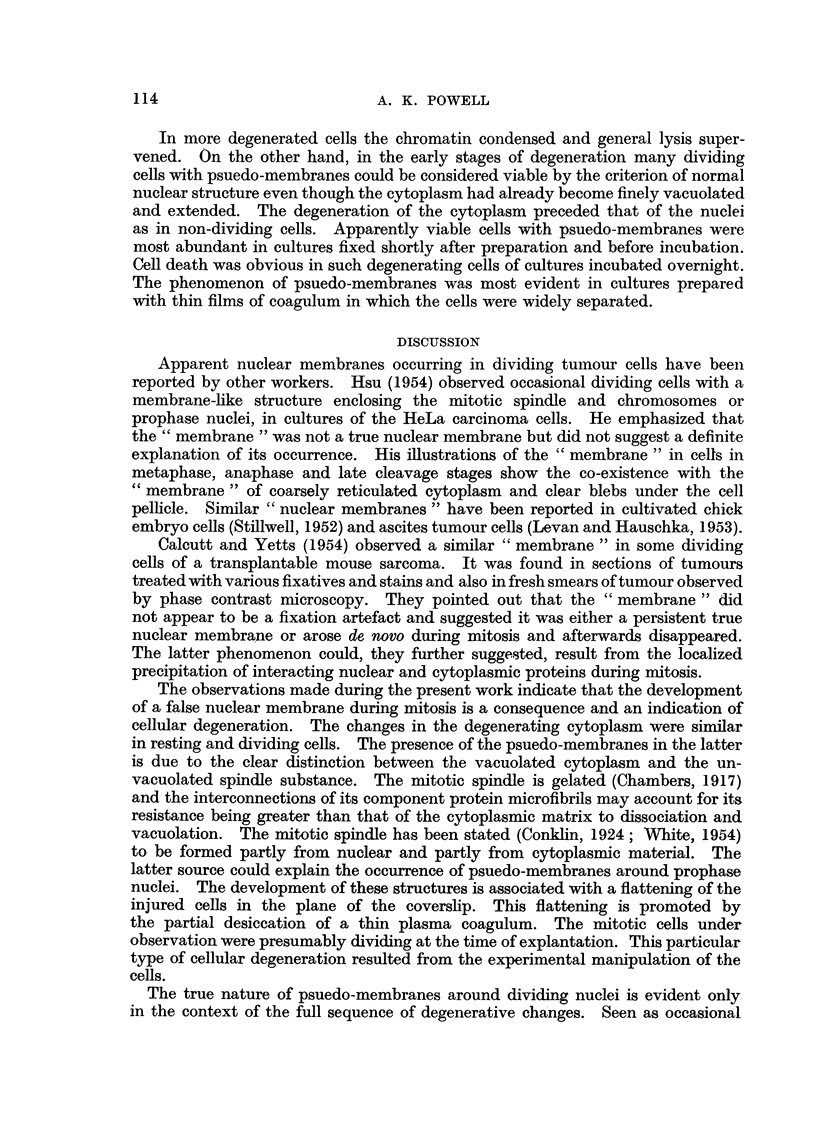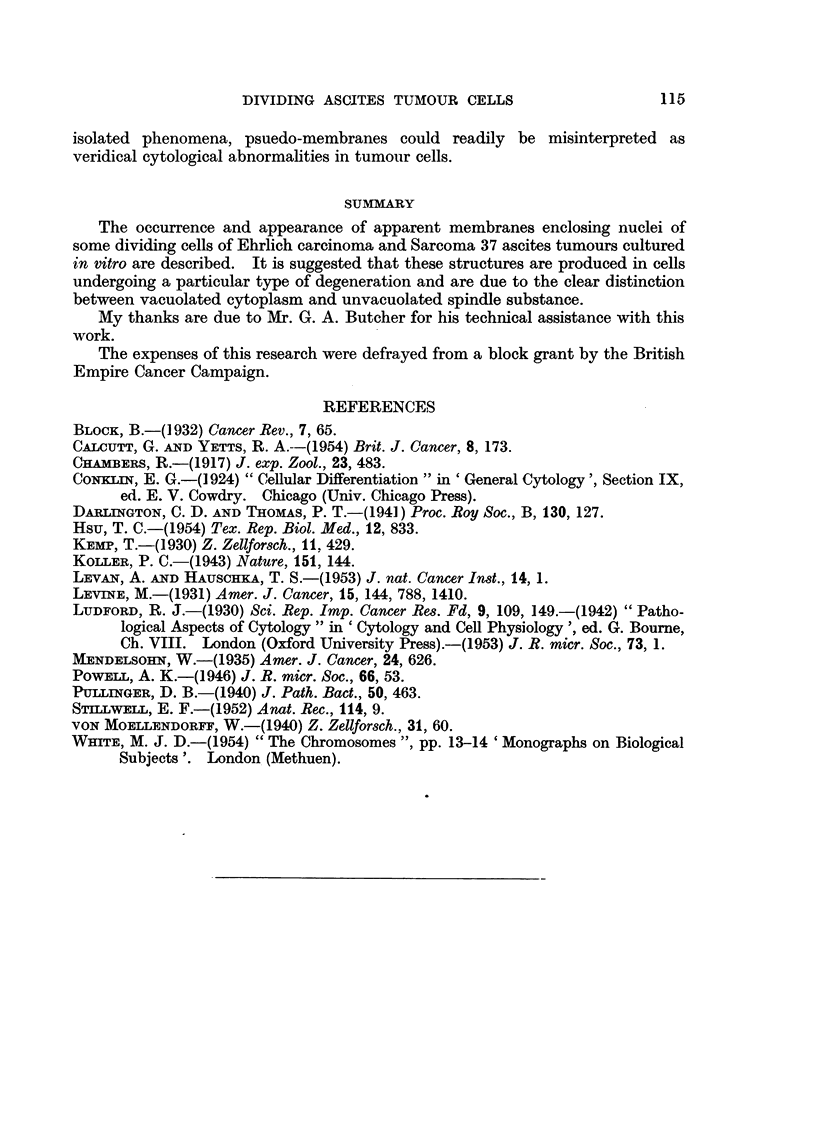# Pseudo-Membranes Enclosing Nuclei of Dividing Ascites Tumour Cells

**DOI:** 10.1038/bjc.1957.16

**Published:** 1957-03

**Authors:** A. K. Powell

## Abstract

**Images:**


### PSUEDO-MEMBRANES ENCLOSING NUCLEI OF DIVIDING ASCITES TUMOUR CELLS

A. K. POWELL

*From the Department of Experimental Pathology, Mount Vernon Hospital, Northwood, Middlesex*

Received for publication January 23, 1957

THE cytological abnormalities of neoplastic cells have been studied by many workers because of their possible significance in carcinogenesis, malignancy and diagnosis. Abnormal mitotic figures, including multipolar spindles (Kemp, 1930 ; von Moellendorff, 1940) and absence of a spindle (Ludford, 1930, 1942), have been reported in tumour cells. They have also been seen in pre-cancerous cells (Block, 1932 ; Mendelsohn, 1935). Disturbances of chromosome segregation have been said to be common in malignant cells (Kemp, 1930 ; Levine, 1931 ; Ludford, 1942). The chromosome numbers of the cells of a single tumour may vary greatly (Ludford, 1930, 1942). Binucleate and multinucleate tumour cells have been reported (Levine, 1931 ; Koller, 1943) and in mouse epidermis during early carcinogenesis (Pullinger, 1940).

Similar aberrations occur in non-neoplastic cells. The mitotic abnormalities observed in malignant cells can be produced in normal cells by a variety of non-carcinogenic and carcinogenic substances (Ludford, 1953). However, strains of malignant cells cultivated *in vitro* (Ludford, 1942) and many tumours *in vivo* are characterized by a high incidence of abnormalities. The structural abnormalities of malignant cells may be regarded as consequences of the malignant state (e.g. Powell, 1946) or as being directly related to the initiation and retention of malignancy. For example, disturbances in the euchromatin-heterochromatin balance suggested by Darlington and Thomas (1941) and Koller (1943) as the initiating cause of malignancy could result from derangements of the spindle and subsequent variations in chromosome number and arrangement.

In view of the debatable significance of cytological abnormalities in relation to the cancer problem it is important to distinguish genuine from false aberrations. During studies upon Ehrlich carcinoma and Sarcoma 37 mouse ascites tumour cells cultivated *in vitro* an apparent membrane-like structure enclosing the dividing nucleus was seen in a small proportion of cells. This structure was found to be associated with a particular sequence of degenerative changes in freshly explanted cells. Seen as an isolated phenomenon this " membrane " might be interpreted

---

EXPLANATIONS OF PLATE

Photomicrographs of normal and degenerating Ehrlich carcinoma ascites cells in plasma cultures.

FIG. 1.—Resting cell, normal.
FIG. 2.—Resting cell, early stage of degeneration.
FIG. 3.—Resting cell, later stage of degeneration.
FIG. 4.—Cell in prophase, degenerated.
FIG. 5.—Cells in metaphase, normal and degenerated.
FIG. 6.—Cell in metaphase, degenerated.
FIG. 7.—Cell in anaphase, normal.
FIG. 8.—Cell in anaphase, degenerated.

1

2

3

4

Powell.

5 6 7 8

as a veridical cellular structure and given an unwarranted significance. For this reason a brief account is given of its appearance and occurrence in the tumour cells studied.

### MATERIAL AND METHODS

False membranes were found in a small proportion of cells of both strains which had been explanted in plasma-embryo extract coagula in coverslip cultures. Freshly collected ascites tumour fluid was diluted with from 9 to 99 parts of a mixture of equal parts of heparinized fowl plasma and ascites tumour plasma. The latter was prepared almost immediately before use by centrifuging fresh ascitic fluid. Homologous ascitic plasma was used for each strain of tumour. Equal volumes of the diluted ascitic fluid containing the tumour cells and either chick or mouse embryo extract prepared with 10 per cent hypotonic Earle's buffered saline solution were mixed and spread in a thin coagulum to prepare the coverslip culture. The cultures were fixed in Heidenhain's Susa mixture and stained with dilute Harris' haematoxylin. The development of false nuclear membranes was readily induced by leaving thinly populated cultures exposed in covered petri dishes at laboratory temperatures for 30 or more minutes before fixation.

### OBSERVATIONS

Four main classes of degenerating ascites tumour cells were found in the cultures. Three of these were distinguished by marked hydropic vacuolation, simple autolytic changes, and changes due primarily to mechanical stress in the coagula, respectively.

Psuedo-membranes were associated with a fourth type of degeneration which was initially distinguished by progressive alterations in the structure of the cytoplasm. In contradistinction to the homogeneous cytoplasm of a healthy cell with its few small fat droplets (Fig. 1), degenerating cytoplasm developed very numerous small vacuoles and appeared reticulated (Fig. 2, 3). The width of the cytoplasm was greatly increased in comparison with the typical narrow zone in healthy cells. With further degeneration the vacuoles enlarged and the cytoplasm became more distended. Finally, the reticulated strands of protoplasm broke into discrete granules and the vacuoles coalesced.

During early stages of this form of degeneration the nuclei retained a normal structure, although it was optically sharper than in healthy cells, and affected cells could be considered viable. Later, the nuclei became pyknotic or lytic. In non-dividing cells vacuolated cytoplasm occupied the cell from the pellicle to the nuclear membrane.

Dividing tumour cells in which the nuclear membranes had dissolved reacted to the early cytoplasmic changes so as to suggest the presence of a persistent nuclear membrane during metaphase, anaphase and telophase stages (cf. Fig. 5–8). In such cells the nuclear region appeared relatively normal. It was occupied by chromosomes embedded in mitotic spindle substance which appeared structurally normal, sometimes showed axial striae and lacked vacuoles. The demarcation between spindle substance and the vacuolated cytoplasm was well delimited but not as sharply defined as a true nuclear membrane. Prophase nuclei of cells in early stages of degeneration were sometimes surrounded by a narrow zone of unvacuolated protoplasm, itself enclosed in a much wider zone of vacuolated cytoplasm (Fig. 4).

In more degenerated cells the chromatin condensed and general lysis supervened. On the other hand, in the early stages of degeneration many dividing cells with psuedo-membranes could be considered viable by the criterion of normal nuclear structure even though the cytoplasm had already become finely vacuolated and extended. The degeneration of the cytoplasm preceded that of the nuclei as in non-dividing cells. Apparently viable cells with psuedo-membranes were most abundant in cultures fixed shortly after preparation and before incubation. Cell death was obvious in such degenerating cells of cultures incubated overnight. The phenomenon of psuedo-membranes was most evident in cultures prepared with thin films of coagulum in which the cells were widely separated.

### DISCUSSION

Apparent nuclear membranes occurring in dividing tumour cells have been reported by other workers. Hsu (1954) observed occasional dividing cells with a membrane-like structure enclosing the mitotic spindle and chromosomes or prophase nuclei, in cultures of the HeLa carcinoma cells. He emphasized that the " membrane " was not a true nuclear membrane but did not suggest a definite explanation of its occurrence. His illustrations of the " membrane " in cells in metaphase, anaphase and late cleavage stages show the co-existence with the " membrane " of coarsely reticulated cytoplasm and clear blebs under the cell pellicle. Similar " nuclear membranes " have been reported in cultivated chick embryo cells (Stillwell, 1952) and ascites tumour cells (Levan and Hauschka, 1953).

Calcutt and Yetts (1954) observed a similar " membrane " in some dividing cells of a transplantable mouse sarcoma. It was found in sections of tumours treated with various fixatives and stains and also in fresh smears of tumour observed by phase contrast microscopy. They pointed out that the " membrane " did not appear to be a fixation artefact and suggested it was either a persistent true nuclear membrane or arose *de novo* during mitosis and afterwards disappeared. The latter phenomenon could, they further suggested, result from the localized precipitation of interacting nuclear and cytoplasmic proteins during mitosis.

The observations made during the present work indicate that the development of a false nuclear membrane during mitosis is a consequence and an indication of cellular degeneration. The changes in the degenerating cytoplasm were similar in resting and dividing cells. The presence of the psuedo-membranes in the latter is due to the clear distinction between the vacuolated cytoplasm and the unvacuolated spindle substance. The mitotic spindle is gelated (Chambers, 1917) and the interconnections of its component protein microfibrils may account for its resistance being greater than that of the cytoplasmic matrix to dissociation and vacuolation. The mitotic spindle has been stated (Conklin, 1924 ; White, 1954) to be formed partly from nuclear and partly from cytoplasmic material. The latter source could explain the occurrence of psuedo-membranes around prophase nuclei. The development of these structures is associated with a flattening of the injured cells in the plane of the coverslip. This flattening is promoted by the partial desiccation of a thin plasma coagulum. The mitotic cells under observation were presumably dividing at the time of explantation. This particular type of cellular degeneration resulted from the experimental manipulation of the cells.

The true nature of psuedo-membranes around dividing nuclei is evident only in the context of the full sequence of degenerative changes. Seen as occasional isolated phenomena, psuedo-membranes could readily be misinterpreted as veridical cytological abnormalities in tumour cells.

### SUMMARY

The occurrence and appearance of apparent membranes enclosing nuclei of some dividing cells of Ehrlich carcinoma and Sarcoma 37 ascites tumours cultured *in vitro* are described. It is suggested that these structures are produced in cells undergoing a particular type of degeneration and are due to the clear distinction between vacuolated cytoplasm and unvacuolated spindle substance.

My thanks are due to Mr. G. A. Butcher for his technical assistance with this work.

The expenses of this research were defrayed from a block grant by the British Empire Cancer Campaign.

### REFERENCES

Block, B.—(1932) *Cancer Rev.*, **7**, 65.
Calcutt, G. and Yetts, R. A.—(1954) *Brit. J. Cancer*, **8**, 173.
Chambers, R.—(1917) *J. exp. Zool.*, **23**, 483.
Conklin, E. G.—(1924) " Cellular Differentiation " in ' General Cytology ', Section IX, ed. E. V. Cowdry. Chicago (Univ. Chicago Press).
Darlington, C. D. and Thomas, P. T.—(1941) *Proc. Roy Soc.*, B, **130**, 127.
Hsu, T. C.—(1954) *Tex. Rep. Biol. Med.*, **12**, 833.
Kemp, T.—(1930) *Z. Zellforsch.*, **11**, 429.
Koller, P. C.—(1943) *Nature*, **151**, 144.
Levan, A. and Hauschka, T. S.—(1953) *J. nat. Cancer Inst.*, **14**, 1.
Levine, M.—(1931) *Amer. J. Cancer*, **15**, 144, 788, 1410.
Ludford, R. J.—(1930) *Sci. Rep. Imp. Cancer Res. Fd*, **9**, 109, 149.—(1942) " Pathological Aspects of Cytology " in ' Cytology and Cell Physiology ', ed. G. Bourne, Ch. VIII. London (Oxford University Press).—(1953) *J. R. micr. Soc.*, **73**, 1.
Mendelsohn, W.—(1935) *Amer. J. Cancer*, **24**, 626.
Powell, A. K.—(1946) *J. R. micr. Soc.*, **66**, 53.
Pullinger, D. B.—(1940) *J. Path. Bact.*, **50**, 463.
Stillwell, E. F.—(1952) *Anat. Rec.*, **114**, 9.
von Moellendorff, W.—(1940) *Z. Zellforsch.*, **31**, 60.
White, M. J. D.—(1954) " The Chromosomes ", pp. 13–14 ' Monographs on Biological Subjects '. London (Methuen).